# Genetic Evaluation of Milk Production Traits in the Serbian Saanen Goat Population

**DOI:** 10.3390/ani15203008

**Published:** 2025-10-16

**Authors:** Krstina Zeljić Stojiljković, Nenad Mićić, Vladan Bogdanović, Radica Đedović, Ivan Pihler, Nenad Stojiljković, Dragan Stanojević

**Affiliations:** 1 Institute for Animal Husbandry, Autoput 16, P.O. Box 23, 11080 Belgrade, Serbia; micicnenad@hotmail.com (N.M.); nstojiljkovic.izs@afrodita.rcub.bg.ac.rs (N.S.); 2 Department of Animal Science, Faculty of Agriculture, University of Belgrade, Nemanjina 6, 11080 Belgrade, Serbia; vlbogd@agrif.bg.ac.rs (V.B.); genrad@agrif.bg.ac.rs (R.Đ.); stanojevic@agrif.bg.ac.rs (D.S.); 3 Department of Animal Science, Faculty of Agriculture, University of Novi Sad, Trg Dositeja Obradovića 8, 21000 Novi Sad, Serbia; ipihler@gmail.com

**Keywords:** total milk yield, heritability, selection, lactation, modeling, EBV

## Abstract

**Simple Summary:**

The estimation of breeding values in the goat population in Serbia is not systematically implemented, resulting in the genetic parameters of economically important milk traits remaining largely unknown. It is well established that the effectiveness of selection depends on the accuracy of heritability; therefore, it is essential to apply modern methods and models for estimating the heritability of milk production traits in goats. In such estimations, as well as in the estimation of breeding values for milk traits in goats, the Sire model is most commonly used in genetic evaluations at the national level. Although traditionally applied, the Sire model has limitations, as it does not fully utilize all available pedigree and performance data. In this study, milk production traits were analyzed in a population of Saanen goats using both the Sire model and the more advanced Animal model. The Animal model, which incorporates information from all known relatives, proved to be more accurate and informative in the estimation of breeding values. The results indicated strong correlations between the two models; however, the Animal model provided more accurate and reliable breeding value estimates, making it more suitable for practical use in breeding programs. These findings underscore the importance of applying more modern and advanced methods in the genetic evaluation of milk production traits in goats, particularly for economically significant traits such as milk yield and composition.

**Abstract:**

Within the framework of this study, a genetic evaluation of milk traits was conducted in the Saanen goat breed. The focus of the research was placed on the application of more advanced models for estimating heritability and breeding values of economically important milk traits. The study included 670 Saanen goats and a total of 2155 lactations between 2010 and 2021 on a single farm located in the Autonomous Province of Vojvodina. The milk production traits included total milk yield per lactation (TMY), milk fat yield (FY), protein yield (PY), and the content of milk fat (MF) and protein (PC). The fixed effects included in the Sire and Animal models were as follows: kidding season, type of kidding, year of kidding, and lactation number. The permanent environmental effect of the doe and the animal’s additive genetic effect were considered as random effects. In the Animal model, the estimated heritability values for the traits were: 0.2216 (TMY), 0.2564 (FY), 0.2556 (PY), 0.3977 (MF), 0.2864 (PC). The heritability estimates obtained using the sire model were slightly higher: 0.2742 for TMY, 0.3256 for FY, 0.3855 for PY, 0.3925 for MF, and 0.3502 for PC. The estimation of breeding values for bucks was performed using both the Sire model and the Animal model. Breeding values for the bucks derived from the two models showed a close relationship, with correlations ranging from 0.85 for TMY to 0.90 for PC. The results of this study confirm that the application of the BLUP-Animal model provides a more accurate estimation of breeding values and represents a reliable basis for the selection of the Saanen goat breed. The findings from this study provide a practical basis for enhancing breeding programs and developing an effective strategy for genetic improvement of milk production in the population.

## 1. Introduction

The importance and demand for goat milk and goat milk products have significantly increased over the past decade, both globally and nationally. The global goat population has been increasing in numbers over the past 30 years [1]. According to FAOSTAT data from 2024, the global goat population is approximately 1.01 billion head [2]. This trend is driven by its favorable organoleptic and nutritional properties for human health compared to cow’s milk, making it suitable for sensitive consumers, including children and individuals with allergies or lactose intolerance [3]. Goat milk is gaining attention due to its higher digestibility and suitability for people who show intolerance to cow’s milk [4]. The smaller diameter of fat globules in goat milk, compared to the milk of other livestock species, contributes to its higher digestibility in humans [5]. Goat milk is often classified as a functional food, as it provides multiple health advantages owing to its high content of medium-chain fatty acids, oligosaccharides, essential amino acids, and easily absorbed minerals [6]. Goats also exhibit an exceptional ability to adapt to diverse and often unfavorable rearing conditions, making them a cost-effective and environmentally sustainable choice for livestock production [7,8].

The Saanen goat breed, originating from Switzerland, is one of the most productive dairy breeds in the world [9]. According to a study carried out in China [10], the Saanen breed tended to produce higher milk yields than Toggenburg and Alpine-Yunshang dual-purpose F1 (AJF1) goats, although these results may depend on environmental and management factors. The governments of several Asian countries have decided to import large numbers of Saanen goats, due to their well-known high milk production potential [11].

Due to its good adaptability, high milk production potential, and favorable composition of milk, the Saanen breed has also found its place in goat farming in the Republic of Serbia. In Serbia, the Saanen breed is one of the two most important commercial dairy breeds (alongside Alpine goats), representing a significant portion of the goat population. This provides a solid foundation for selection and the improvement of production traits within this branch of livestock farming.

Milk yield and quality in all ruminant species are the result of a complex interaction between genetic and non-genetic factors [12]. To achieve genetic progress in selection programs, it is essential to accurately identify and incorporate these factors into statistical models for the estimation of genetic parameters and breeding values. Accurate estimation of genetic parameters and selection of economically important traits requires proper modeling of all relevant fixed and random factors.

According to the findings of a previous study [13], the month of kidding (with higher yields in spring than in autumn and winter), lactation number, and litter size are significant factors affecting milk production in goats. A higher number of kids and increased parity are associated with greater total milk production. Another study [14], indicates that age represents a major determinant of milk yield and composition in Saanen goats. Milk yield significantly increased with the age of the goats. Regarding milk composition and quality, the study showed that two-year-old (first-parity) and three-year-old (second-parity) goats did not differ significantly in terms of fat, total solids, protein, and lactose content, whereas four-year-old goats (after the third kidding) exhibited a notably higher fat content.

In terms of composition, goat milk typically contains around 12.2% dry matter, of which fat represents close to 3.8%, protein 3.5%, lactose 4.1%, and mineral substances (ash) about 0.8% [15]. Similarly, variation in milk composition in cattle was affected by several factors, including breed, lactation stage, herd, parity order, somatic cell count (udder health), altitude, and season of the year [16]. Consequently, fat content ranges from 2.61% to 5.03%, protein from 3.16% to 3.40%, lactose from 4.74% to 5.12%, and minerals from 0.70% to 0.76%. Such a comparison highlights the unique properties of goat milk that can make it a suitable alternative for specific dietary needs.

Genetic improvement of production traits and accurate estimation of breeding values form the foundation of a successful animal selection program. Traditionally, this process has been based on the identification of animals and the measurement of their morphological and production characteristics, such as milk yield, fat and protein content, body measurements, and similar traits [17]. Nevertheless, these approaches are limited in accuracy, particularly in the early life stages of animals and when production data are unavailable.

Similarly, other authors such as [18,19,20,21] emphasize the importance of including the animal’s additive genetic effect and the permanent environmental effect as random factors in models for estimating genetic parameters. Such models, like the BLUP-Animal model, provide more accurate estimation of breeding values because they account for relatives as well as all available information about the individual and its relatives.

Improved accuracy in the estimation of genetic potential can be achieved by incorporating detailed pedigree data, kinship, and genetic variability within the population. Modern approaches that integrate phenotypic information with genetic and genomic data enable significantly more reliable estimation of genetic parameters and breeding values [22,23]. These data not only increase the accuracy of selection but also enable earlier identification of superior animals, thereby contributing to faster genetic progress within the selected population.

The selection and breeding of goats in the Republic of Serbia is still largely based on the assessment of phenotypic characteristics of animals. Classification and selection of animals for further reproduction are based on body development, condition, and overall appearance of the individuals. Additionally, milk yield recording is carried out on some farms, conducted in accordance with ICAR standards using the AT4 method [24], and serves as one of the criteria for selection.

Nevertheless, the application of modern genetic and statistical methods for the estimation of breeding values of milk traits in does and bucks has not yet been established in practical use within Serbia. Such estimation would enable more precise identification of genetically superior animals, thereby accelerating genetic progress and improving herd productivity. The implementation of methods such as the BLUP-Animal model (Best Linear Unbiased Prediction—Animal model), combining available pedigree data and production records, represents an important step towards enhancing breeding programs in Serbian goat farming.

Estimation of heritability and breeding values of animals represents a key element in the development and improvement of breeding programs, as it enables selection based on genetic potential rather than solely on phenotypic characteristics. Research on the estimation of genetic parameters and breeding values in goat farming in the Republic of Serbia has so far been relatively limited, highlighting the need for a more systematic approach to this topic. Accordingly, the objectives of this study were threefold: (1) to analyze the effects of various fixed factors (kidding season, year, lactation number, and type of kidding) on key milk production traits in the Saanen goat breed, including total milk yield (TMY), milk fat yield (FY), protein yield (PY), and the fat (MF) and protein content (PC) in milk; (2) to estimate genetic parameters, primarily heritability, for these traits based on models incorporating the animal’s additive genetic effect and permanent environmental effect; and (3) to evaluate the breeding values of bucks using both the Sire model and the Animal model to obtain a more reliable ranking of bucks according to their genetic superiority for milk traits.

This study aims to contribute to the understanding of the genetic basis of production traits in goats under local conditions and to serve as a foundation for the implementation of modern breeding methods in Serbian goat farming practices.

## 2. Materials and Methods

### 2.1. Population Description and Data Collection

The data for this study were collected from a commercial farm in the Autonomous Province of Vojvodina, Serbia, and consisted of 670 Saanen does that kidded between 2010 and 2021. The Saanen goat is a high-yielding dairy breed and represents one of the most prevalent commercial dairy breeds in the Republic of Serbia. The choice of this population allowed consistent long-term monitoring of production traits under typical farming conditions in Serbia, thereby ensuring that the results are directly applicable to practical breeding and management programs.

The dataset used for the analysis contained information on 2155 completed lactations recorded during the specified period. The average lactation length was 289 days, which is consistent with expectations for this breed under intensive management conditions.

Lactation data were collected within the framework of official milk recording, conducted by the primary breeding organization responsible for the territory in which the farm is located. This organization provided access to the data for this study, in accordance with the official breeding program and established ethical guidelines.

The animals were housed in group pens accommodating 40 to 50 individuals and reared under an intensive management system without access to pasture. Grazing was not part of the production practice, which allowed for a consistent feed intake and greater control over nutritional conditions. Milking was performed using an automatic milking machine twice daily—morning and evening—at average intervals of 12 h, thereby ensuring optimal conditions for stable production and regular data collection.

### 2.2. Statistical Models and Genetic Evaluation Methods

The significance of fixed effects on milk production traits—total milk yield (TMY), milk fat yield (FY), protein yield (PY), and the content of milk fat (MF) and protein (PC) in milk—was determined using the General Linear Model (GLM) procedure within the SAS software package, version 9.4. [25]. The selection of fixed effects to be included in the variance components estimation model was performed using a stepwise procedure, with the statistical significance of individual effects serving as the selection criterion.

The fixed factors included in the analysis were kidding season, kidding type, kidding year, and lactation number, while lactation length was incorporated as a regression effect (Table 1). This model structure allowed for the elimination of non-genetic systematic effects, thereby improving the accuracy of genetic parameter estimation. The analysis of non-genetic effects on TMY, as well as the estimation of least square means (LSM) for different levels of fixed factors, was performed using the GLM procedure within the same statistical software. Since the kidding season did not show a significant effect on fat and protein content, this factor was excluded from the analyses. Consequently, the AM 4 and Sire Model 2 were applied for these traits.

The random effect of the permanent environment of each doe included all lactations of the same animal and was evaluated individually, aiming to account for persistent, non-genetic environmental influences that recur across multiple lactations of the same animal.

In order to estimate and compare breeding values obtained from two different statistical models—the Sire model and the Animal model—it was first necessary to estimate variance components for all evaluated milk production traits. The estimation of these components forms the basis for separating genetic and environmental variability within the population, and for obtaining the parameters required to calculate heritability and breeding values.

For the purpose of consistent comparison, identical linear mixed models with the same fixed, regression, and random effects were applied in both cases to ensure the comparability of results and to eliminate methodological differences that could influence the estimation of breeding values.

Variance components were estimated using the restricted maximum likelihood (REML) method, utilizing the specialized software package VCE-6 [26] which is designed for the analysis of variance components in multitrait genetic models. Subsequently, the estimation of breeding values (EBV) for animals, specifically bucks, was performed using the BLUP methodology within the PEST software package [27].

Heritabilities for the examined traits were estimated using the following linear mixed models:

(1) $$Y_{\mathrm{ijklmn}}\;=\;S_{i}\;+\;T_{j}\;+\;G_{k}\;+\;{\mathrm{RB}}_{l}\;+\;b_{1m}(x_{\mathrm{ijklmn}}-\bar{X})+o_{\mathrm{ijklmn}}+e_{\mathrm{ijklmn}}$$

(2) $$Y_{\mathrm{ijklmn}}=T_{j}+G_{k}+{\mathrm{RB}}_{l}+b_{1m}(x_{\mathrm{ijklmn}}-\bar{X})+o_{\mathrm{ijklmn}}+e_{\mathrm{ijklmn}}$$

Sire Model (1 and 2), where

Si—fixed effect of season (i = 1—March, April, May = spring; 2—June, July, August = summer; 3—September, October, November = autumn; 4—December, January, February = winter);

Tj—fixed effect of type of kidding (j = 1, 2, 3, 4);

Gk—fixed effect of year of kidding (k = 2010, 2011, …, 2021);

RBl—lactation number (l = 1, 2, …, 10);

b1m(xijklmn—$\bar{X}$)—linear regression effect of lactation length;

oijklmn—random effect of sire;

eijklmn—residual.

(3) $$Y_{\mathrm{ijklmno}}=S_{i}+T_{j}+G_{k}+{\mathrm{RB}}_{l}+b_{1m}(x_{\mathrm{ijklmno}}-\bar{X})+p_{\mathrm{ijklmno}}+a_{\mathrm{ijklmno}}+e_{\mathrm{ijklmno}}$$

(4) $$Y_{\mathrm{ijklmno}}=T_{j}+G_{k}+{\mathrm{RB}}_{l}+b_{1m}(x_{\mathrm{ijklmno}}-\bar{X})+p_{\mathrm{ijklmno}}+a_{\mathrm{ijklmno}}+e_{\mathrm{ijklmno}}$$

Animal Model (3 and 4), where

Si—fixed effect of season (i = 1—March, April, May; 2—June, July, August; 3—September, October, November; 4—December, January, February);

Tj—fixed effect of type of kidding (j = 1, 2, 3, 4);

Gk—fixed effect of year of kidding (k = 2010, 2011, …, 2021);

RBl—lactation number (l = 1, 2, …, 10);

b1m(xijklmno—$\bar{X}$)—linear regression effect of lactation length;

pijklmno—random effect of the doe’s permanent environment affecting her successive lactations;

aijklmno—direct additive genetic effect of the animal;

eijklmno—residual.

The percentage contribution of individual variance components to the total phenotypic variance was determined using the REML. To ensure reliable estimation of additive genetic variance, it was necessary to construct a pedigree relationship matrix. For this purpose, a pedigree file was created encompassing all animals included in the analysis, as well as their known ancestors, based on the available pedigree information

The relatedness matrix enabled the adequate incorporation of genetic relationships between individuals into the model, which is a prerequisite for accurate estimation of genetic variability within the population. Based on the obtained variance values, heritabilities were calculated according to the following formula:

$$h^{2}=\frac{\sigma_{a}^{2}}{\sigma_{p}^{2}}$$

where

ℎ^2^—the heritability of the trait in the examined population;

$\sigma_{a}^{2}$—additive genetic variance (the genetic potential of an animal that can be transmitted to its progeny);

$\sigma_{p}^{2}$—phenotypic variance (the variation in phenotypes observed within the population).

Pedigree information of the animals, which is essential for the construction of the pedigree file and the formation of the relatedness matrix, was analyzed using the CFC software package v.1.0. [28]. This software enabled a detailed pedigree analysis, including the determination of pedigree depth, identification of base animals, as well as verification of the completeness and connectedness of pedigree data. The pedigree served as the basis for constructing the additive genetic relatedness matrix of the animals in the analyzed population, which is used in BLUP analysis to estimate additive genetic variance and breeding values.

The content and structure of the pedigree used for estimating the heritability of milk production traits in goats are presented in Table 2, which provides information on the number of individuals with production records, the number of ancestors, the percentage of base animals, as well as the distribution of sires and dams within the analyzed population. These data ensured adequate coverage of genetic information within the population and contributed to the accuracy of the genetic parameter estimates.

In order to compare the ranking results of bucks based on different statistical models, a rank correlation analysis was conducted for the estimated breeding values obtained using the BLUP Animal Model (AM) and the BLUP Sire Model (SM). Ranking of animals based on their breeding values has practical importance in selection programs, as it directly influences the choice of parents for the next generation. Therefore, comparing the outcomes of different models is essential for assessing their reliability under practical conditions.

To assess the agreement between breeding value estimates obtained using the animal and sire models, Spearman’s rank correlation coefficient was applied. This non-parametric statistical method measures the degree of agreement in the ranking order of individual animals between two independent datasets and is particularly suitable in situations where values obtained from different models—with the same data structure but different assumed distributions of errors and effects—are being compared.

The rank correlation was calculated for all bucks included in the analysis, i.e., for all sires within the population for whom breeding values were estimated in both models. The formula used for calculating Spearman’s rank correlation coefficient is as follows:

$$r_{s}=1-\frac{6\sum d^{2}}{\left[n\left(n^{2}-1\right)\right]}$$

where

*r_s_*—value of Spearman’s rank correlation coefficient;

*d*^2^—square of the difference between individual ranks;

*n*—number of ranked animals.

## 3. Results

### 3.1. Phenotypic Overview and Systematic Effects

The average milk yield per lactation in the examined population of Saanen goats was 697.59 kg, which indicates a relatively high production under an intensive management system for this breed. The average fat yield was 22.80 kg, while the average protein yield was 19.90 kg, suggesting a relatively consistent milk composition within the observed herd.

Regarding the composition of milk, the average fat content was 3.25%, while the average protein content was 2.85%. These values indicate that the milk composition falls within the range generally considered suitable for dairy processing, according to published standards for milk quality.

Descriptive statistics for all observed traits, including mean values, minimums, maximums, standard deviations, and coefficients of variation (CV), are presented in Table 3. The coefficients of variation indicate a moderate to high degree of phenotypic variability, which provides a basis for successful selection, i.e., identification of animals with the highest genetic potential for high milk production.

In the Saanen goat population, most milk production traits were significantly influenced by the fixed effects included in the model. The statistical significance of individual factors, including season of kidding, type of kidding, year of kidding, lactation number, and lactation length, is shown in Table 4.

For the trait TMY, all the factors listed above were statistically significant, with significance levels ranging from *p* < 0.05 to *p* < 0.001. The greatest contributors to the variability of this trait were the kidding year and the regression effect of lactation length. The coefficient of determination (R^2^) for this trait was 0.427, indicating that the included factors explain approximately 43% of the total phenotypic variance.

A similar pattern of significance was observed for fat yield (FY) and protein yield (PY), with R^2^ values of 0.406 and 0.405, respectively. For fat content (FC) and protein content (PC), kidding season did not have a statistically significant effect (*p* > 0.05), and therefore, this factor was not included in the model for estimating variance components of these traits. Since kidding season did not show a significant effect on fat and protein content, this variable was excluded from the analyses, which is the reason why AM 4 and Sire model 2 were applied for these traits.

### 3.2. Estimation of Genetic Variance Components and Breeding Values

The estimated heritability values for five milk production traits (TMY, FY, PY, MF, and PC) were obtained using two models: the Animal model and the Sire model. In the Animal model, the heritability values were 0.2216 for TMY, 0.2564 for FY, 0.2556 for PY, 0.3977 for MF, and 0.2864 for PC. In the Sire model, the estimated values were slightly higher: 0.2742 for TMY, 0.3256 for FY, 0.3855 for PY, 0.3925 for MF, and 0.3502 for PC.

Complete values of additive genetic variance (σ^2^ₐ), permanent environmental variance (σ^2^ₚ), error variance (σ^2^ₑ), as well as total phenotypic variance (σ^2^ₚₕ), are presented in Table 5 and Table 6. The percentages of the total phenotypic variance originating from the permanent environment and the residual (error) were also calculated, which contributed to a more accurate interpretation of the obtained results.

In the animal model, the permanent environmental effect of the goat (p^2^) accounted for a considerable proportion of the total variance for all traits (Table 5), highlighting the important influence of environmental factors recurring in the same individuals across multiple lactations.

In this study, high Spearman’s rank correlation coefficients (0.847–0.904) were observed between the breeding values for milk production traits estimated using the Animal model and the Sire model (Table 7). The highest concordance in the ranking of bucks was observed for the PC trait, whereas the lowest, though still high, was recorded for TMY.

Based on the EBV in the Animal Model, buck 7410 is ranked highest for TMY. The daughters of this buck produced on average 102 kg more milk compared to the population average, making him the most desirable animal for selection in terms of milk yield (Supplementary Materials S1). The same buck maintained the leading position in the Sire Model, although with a slightly lower estimate (79 kg above average), which is consistent with the expectation that the Animal Model provides more accurate and higher values due to the use of all available pedigree information.

**Figure 1:**
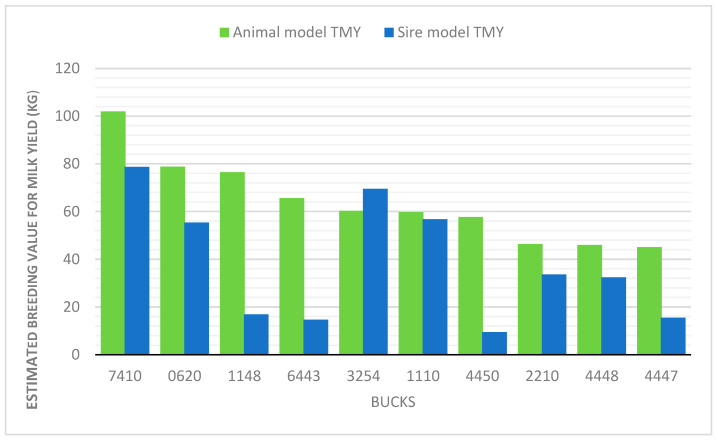
Comparison of the estimated breeding values (EBV) for milk yield (kg) of the top 10 bucks using the Animal and Sire models.

Considering the top twenty bucks in both models, it is noticeable that some animals are consistently ranked high in both, indicating their stable genetic superiority (Figure 1). For example, buck 3254 ranks 5th in the Animal Model and 2nd in the Sire Model, and is characterized by very high values for fat and protein content (Supplementary Materials S1). Similarly, buck 1148 is highly ranked in both models for milk protein content, making him an important candidate for selection aimed at improving the qualitative traits of milk.

On the other hand, certain differences in buck rankings between the models are also observed. For example, buck 6450 ranks 13th for TMY in the Animal model, but only 16th in the Sire model. Nevertheless, he shows exceptionally high values for FY and PY in both models (e.g., PY = 2.95 and 4.76, respectively), indicating his particular value when the focus is on quality rather than solely on the quantity of milk produced. This example illustrates the importance of evaluating multiple traits in parallel, as some bucks may excel in specific milk components.

## 4. Discussion

### 4.1. Phenotypic Characterization and Influence of Non-Genetic Factors on Milk Production Traits

The average values obtained for milk yield and its components in this study are lower compared to the results reported by [29] in a study conducted in Canada, based on a sample of 3434 first-lactation Saanen goats, the average TMY was found to be 921 kg, with 32.9 kg of FY and 28.6 kg of FY, at higher fat and protein content values (3.6% and 3.1%, respectively). The average values obtained in this study (698 kg TMY, 23 kg FY, 20 kg PY; MF = 3.25%, PC = 2.85%) are significantly lower compared to the Canadian sample. These differences are expected considering the longer lactation length (305-day lactation) reported for the Saanen population in Canada (1119 kg TMY), as documented in the study by [30] for Canada and other countries.

On the other hand, the results of this study are similar to the values reported by [20] for Saanen goats in New Zealand, where the average lactation length was 226.4 days, and the milk yield corrected to 305 days was 727.9 kg, with 24.4 kg of milk fat and 22.2 kg of protein. Similarly, Ref. [31] also reported considerably lower values in a study conducted on 1279 lactations of Saanen and Alpine breeds, with a lactation length of 270 days, where the average milk yield was 440.87 kg, milk fat 15.26 kg, and protein 13.25 kg. Compared to these studies, the results obtained in this research show a moderate level of production, with potential for further genetic improvement, given the existing variability and stable milk composition. The differences in results between the cited studies may be due to variations in management conditions, feeding practices, lactation length, milking systems, as well as the level of genetic potential within the observed populations.

The analysis of fixed effects included in the model for estimating variance components of milk production traits in the Saanen goat breed provided results that were both statistically significant and biologically meaningful. For TMY, FY, and PY, all examined factors showed significance at varying levels (*p* < 0.05 to *p* < 0.001). This aligns with the established biological understanding of lactation, where factors such as year of kidding, type of kidding, lactation number, and lactation length notably affect productivity.

The greatest influence on the variability of the observed traits was exerted by the year of kidding and the regression effect of lactation length. The inclusion of biologically justified fixed effects contributed to the relatively high coefficient of determination values for production traits (R^2^ = 0.40). This confirms the correctness of the model choice and highlights the importance of an adequate selection of fixed effects when estimating genetic parameters and breeding values.

The results obtained in this study indicate a significant influence of multiple fixed factors on the productive traits of Saanen goats. The year of kidding, type of kidding, lactation number, and lactation length are statistically significant factors in explaining the variance in milk yield, are fully consistent with results from larger studies conducted on Saanen goats. In an Australian study based on 940 lactations, all of the listed factors showed a significant effect on total milk yield throughout all stages of lactation [13]. Similarly, an analysis of 2997 lactations conducted in Belgium revealed that fixed effects account for up to 52% of the phenotypic variance, while variations in fat and protein percentages remain modest and are often of limited biological significance [32].

Lactation number emerged as a particularly important factor, which is consistent with the findings of [33], who reported that the highest milk yield in the Saanen breed was achieved during the fifth lactation (809.62 kg), while the lowest yield was recorded in the first lactation (661.37 kg). These results confirm that the productive potential of goats changes significantly with parity, which has practical implications for selection and the extension of the animal’s productive lifespan.

Furthermore, the effect of kidding type was also statistically significant, aligning with the data reported by the same authors, who showed that goats with triplet births produced an average of 798.06 kg of milk per lactation, which is significantly higher compared to goats with single or twin kids. This finding supports the use of kidding number as a potential indirect selection criterion in the assessment of milk yield. Similarly, Ref. [13] from Australia report that, in commercial Saanen goats, litter size significantly affects milk production, with goats bearing twins or triplets achieving substantially higher milk yields compared to those with single kids. This further confirms our conclusion that kidding type can be a valuable selection criterion. These findings further validate the biological rationale for using kidding type (i.e., the number of kids per kidding) as a variable in models for estimating genetic parameters in goats, with the aim of selecting for high milk production.

On the other hand, kidding season did not have a significant effect on the fat and protein content of milk, indicating that the composition of the milk remains relatively stable regardless of the time of year. This may be attributed to the intensive farming system with controlled feeding and housing conditions, which reduces seasonal variability. Accordingly, for the percentage traits of fat and protein content, certain fixed effects, such as kidding season and lactation length, did not show statistical significance (*p* > 0.05), consistent with the findings of [34,35,36]. This result is expected, as these traits are less susceptible to short-term environmental changes and generally exhibit a more stable phenotypic expression, which is often accompanied by higher heritability. Lower values of the coefficient of determination (R^2^ = 0.15–0.25) for fat and protein percentages are also common for percentage traits.

Overall, the obtained results support previous research and confirm the importance of including relevant fixed effects in statistical models when estimating genetic parameters. The inclusion of significant effects not only enhances the accuracy of genetic evaluation but also contributes to a better understanding of the productive potential within the analyzed population.

### 4.2. Additive Genetic Variance and Breeding Value Prediction

The estimated heritability values in this study were consistent with the results reported by other authors, such as [12,20,37,38] indicating the reliability of the model and data used in the analysis. The values obtained in this study fall within a moderate range and confirm that milk production traits in goats exhibit moderate to high heritability. This is consistent with findings from research on New Zealand dairy goats, where heritability estimates were 0.25 for milk yield, 0.24 for milk fat, and 0.24 for protein, indicating significant genetic potential and enabling successful selection for milk production traits in goats [39].

On the other hand, some authors, such as [18,29,31,40]) have reported lower heritability values for these traits, TMY, FY, and PY (respectively, 0.13, 0.19, and 0.14), but similar for MF and PC (0.36 and 0.33). In contrast, Ref. [41] reported higher values (e.g., up to 0.64 for PC), which may be attributed to differences in management conditions, breed composition, data volume, and methodology.

Differences in the estimated heritability values between the Sire and Animal models are expected and methodologically justified. Specifically, the Animal model (BLUP-AM) incorporates information from all known relatives in the pedigree (including not only sires but also dams, siblings, offspring, and other relatives), thereby utilizing a larger dataset and achieving more accurate genetic evaluations. In contrast, the Sire model relies on a limited set of information, primarily the offspring of sires, which results in less precise and potentially biased estimates.

Although heritability estimates in the Animal model are often slightly lower, this is a result of reduced bias due to better control of fixed and random effects. At the same time, these estimates are statistically more stable and genetically more informative, as they reflect the true genetic variability within the population. This has been confirmed in numerous studies. For example, Ref. [42] demonstrated that the BLUP Animal model provides lower but more reliable heritability estimates in dairy cattle compared to the Sire model. Results from studies such as [43,44] confirm that the Animal model generates more stable genetic evaluations due to a more comprehensive utilization of available data and effects.

The high proportion of permanent environmental effects in the total phenotypic variance (31.7% for total milk yield and 26.7% for fat content) confirms that factors such as nutrition, health status, and condition during lactation have a lasting and significant impact on productivity. This finding is consistent with studies using random regression models in Norwegian goats, where the permanent environmental effect plays a significant role in the total phenotypic variance, especially during the early stages of lactation. It is also supported by meta-analyses of genetic parameters in dairy goats, which show that permanent environment often accounts for a substantial portion of the variance, particularly for milk yield traits [29,45].

The results of the model comparison confirm that both the Sire and Animal models produce similar trends in breeding value estimation, as evidenced by high Spearman rank correlation coefficients (0.85–0.90). Similar agreement has been reported by other authors [46], further supporting the reliability of this approach.

Although the Sire model has been widely used in previous studies and some practical applications, it is limited because it incorporates information only about sires, neglecting other relatives. In contrast, the BLUP Animal model utilizes complete pedigree data, including parents, offspring, and other relatives, which increases the accuracy of breeding value estimation by an average of 2–5% compared to the Sire model and reduces prediction bias [42,46,47]. Differences between the models are expected and consistent with both theory and practice.

The comparative analysis of these two models further confirms the higher accuracy of the Animal Model, as it incorporates complete information on pedigree and phenotypic values of all relatives, not just sires. Although the correlations between the models are high (0.85–0.90), the ranking of individual animals differs, which can have practical implications in the selection process.

In a Danish study on Holstein cows, the high rank correlation (0.95–0.97) between EBVs from the Animal and Sire models confirms their general agreement, although the Animal model consistently provides higher reliability and accuracy in breeding value assessments [46]. Similarly, the same authors note that the Animal model is considered the standard because it maximizes the correlation between the estimated and true genetic values while minimizing the prediction error variance.

The top-ranked bucks, such as 7410 and 3254, consistently demonstrated superiority in both methods, indicating their high genetic potential for milk yield and the ability of both models to identify it. Conversely, the same bucks occupied the bottom ranks in both models, further confirming the reliability of the obtained estimates and the applicability of the results in selection programs.

Based on the obtained results, it can be concluded that, when pedigree and production data are available, the recommendation is to use the Animal model in selection practice, as it is more accurate and informative. This enables better ranking of animals and optimal selection of parents for the genetic improvement of milk production traits in the Saanen goat breed.

In conclusion, and in line with the objective of this study, it is important to emphasize that current global trends in goat milk production and breeding, together with climate change, have created a need for the development of new breeding systems and methods that can enhance milk yield while simultaneously ensuring animal welfare, resilience, efficiency, and health [48,49].

One of the future goals to strive for is the implementation of genomic selection, which is recognized as one of the most important tools for improving the accuracy of genetic evaluations [50]. Genomic selection is based on predicting the genomic value of animals by linking production data in a reference population that has been previously genotyped and phenotyped. Recent studies, [51] report that countries around the world have begun implementing genomic selection in various populations of dairy goat breeds, such as Saanen and Alpine, including France [30,38,52,53,54], Spain [23], Canada [29], and New Zealand [20].

## 5. Conclusions

This study represents one of the first systematic investigations of genetic parameters and the estimation of breeding values in the Saanen goat population in the Republic of Serbia. The obtained results highlight the importance and justification of applying the BLUP-Animal model for the estimation of breeding values for milk production traits. Although it requires more advanced statistical and computational processing, its application would significantly contribute to the improvement of selection practices. Establishing efficient selection systems for the Saanen goat breed, as well as for other breeds and production groups in Serbia, should be based on the identification and systematic use of genetically superior individuals in reproduction. The results of this study may serve as a basis for further improvement of breeding programs, as well as for the development of a national strategy for the genetic enhancement of dairy traits. Future progress can be achieved through the integration of classical quantitative genetic methods with modern molecular techniques, such as SNP chips, which enable the application of genomic selection. This integrated approach would facilitate a more precise evaluation of genetic potential and contribute to accelerated and more reliable genetic improvement within domestic goat populations.

## Figures and Tables

**Table 1:** Effects included in the models for heritability estimation.

Factors	AM-Model 1	AM-Model 2	Sire-Model 3	Sire-Model 4
Kidding season	F	-	F	-
Kidding type	F	F	F	F
Kidding year	F	F	F	F
Lactation number	F	F	F	F
Lactation length	R	R	R	R
Permanent environment of the doe	S	S	-	-
Additive genetic effect of the doe/buck	S	S	-	-
Random effect of the sire	-	-	S	S

F-Fixed effect; R-Regression effect; S-Random effect.

**Table 2:** Pedigree structure for heritability estimation of milk production traits in Saanen goats.

Pedigree Data	N
Total number of individuals in the pedigree	928
Number of individuals with production records	670
Number of ancestors in the pedigree	258
Base animals in the pedigree (%)	24.68
Number of sires in the pedigree	58
Number of dams in the pedigree	466

**Table 3:** Descriptive statistics for milk production traits in Saanen goats.

Trait	$\bar{X}$	Min	Max	SD	CV (%)
TMY, kg	697.59	32.00	1450.00	193.37	27.72
FY, kg	22.80	2.74	48.80	6.67	29.27
PY, kg	19.90	1.37	39.40	5.37	27.01
MF, %	3.25	2.00	5.09	0.40	12.17
PC, %	2.85	2.08	3.90	0.23	8.11
LL, day	289.87	11.00	409.00	33.52	11.56

TMY—total milk yield per lactation; FY—milk fat yield; PY—milk protein yield; MF—content of milk fat; PC—content of milk protein; LL—lactation length.

**Table 4:** Statistical significance of the effects included in the fixed model for determining variance components of the studied traits.

Factors						
Trait	Kidding Season	Type of Kidding	Year of Kidding	Parity Number	Linear Regression Effect of Lactation Length	R^2^
TMY	2.98 *	9.32 ***	44.92 ***	14.05 ***	495.66 ***	0.42701
FY	5.03 **	3.52 *	51.43 ***	8.06 ***	452.26 ***	0.40584
PY	6.00 **	6.64 **	54.04 ***	11.88 ***	483.66 ***	0.40481
MF	-	5.46 **	28.05 ***	3.67 ***	1.15 ^ns^	0.14662
PC	-	8.64 ***	44.63 ***	2.85 *	0.28 ^ns^	0.25584

TMY—total milk yield per lactation; FY—milk fat yield; PY—milk protein yield; MF—content of milk fat; PC—content of milk protein; ns = *p* > 0.05; * = *p* < 0.05; ** = *p* < 0.01; *** = *p* < 0.001; R^2^-Coefficient of determination.

**Table 5:** Absolute and relative contribution of random effects to the total phenotypic variance for milk production traits of Saanen goats in the Animal model.

Trait	*σ* ^2^ *_a_*	*σ* ^2^ *_p_*	*σ* ^2^ *_e_*	*σ* ^2^ *_ph_*	*h* ^2^ * ± SE*	*p* ^2^	*e* ^2^
TMY	4872.71	6964.93	1015.00	21,988.40	0.2216 ± 0.026	0.3167	0.4616
FY	7.19	5.36	15.50	28.10	0.2564 ± 0.027	0.1910	0.5525
PY	4.70	4.90	8.78	18.40	0.2556 ± 0.025	0.2668	0.4776
MF	0.06	0.02	0.07	0.10	0.3977 ± 0.045	0.1215	0.4807
PC	0.01	0.01	0.02	0.00	0.2864 ± 0.051	0.1874	0.5261

TMY—total milk yield per lactation; FY—milk fat yield; PY—milk protein yield; MF—content of milk fat; PC—content of milk protein; *σ*^2^*_a_*—additive genetic variance; *σ*^2^*_p_*—variance of the permanent environmental effect of the goat; *σ*^2^*_e_*—error variance (residual); *σ*^2^*_ph_*—phenotypic variance; *h*^2^—heritability estimate; *SE*—standard error of heritability; *p*^2^—estimate of the permanent environmental effect of the goat; *e*^2^—estimate of the error (residual).

**Table 6:** Absolute and relative contribution of random effects to the total phenotypic variance for milk production traits of Saanen goats in the sire model.

Trait	*σ* ^2^ *_o_*	*σ* ^2^ *_e_*	*σ* ^2^ *_ph_*	*h* ^2^	*e* ^2^
TMY	1636.48	22,236.00	23,872.50	0.2742 ± 0.076	0.9314
FY	2.42	27.40	29.80	0.3256 ± 0.104	0.9185
PY	1.90	17.80	19.70	0.3855 ± 0.115	0.9036
MF	0.01	0.10	0.10	0.3925 ± 0.104	0.9018
PC	0.00	0.00	0.00	0.3502 ± 0.106	0.9124

TMY—total milk yield per lactation; FY—milk fat yield; PY—milk protein yield; MF—content of milk fat; PC—content of milk protein; *σ*^2^*_o_*—random sire effect; *σ*^2^*_e_*—error variance (residual); *σ*^2^*_ph_*—phenotypic variance; *h*^2^—heritability estimate; *e*^2^—estimate of the error (residual).

**Table 7:** Spearman’s rank correlation coefficients between EBV using the animal model and the sire model.

Trait				
TMY	FY	PY	MF	PC
0.8476	0.8698	0.8861	0.8716	0.9044

TMY—total milk yield per lactation; FY—milk fat yield; PY—milk protein yield; MF—content of milk fat; PC—content of milk protein.

## Data Availability

Data used in this research are available from the corresponding authors on request.

## Supplementary Materials

The following supporting information can be downloaded at: https://www.mdpi.com/article/10.3390/ani15203008/s1. Table S1: Breeding value of bucks for milk production traits estimated using the animal model and the sire model (top 20 ranked).
