# Supplementary material for: Genetic Evaluation of Milk Production Traits in the Serbian Saanen Goat Population
Source: Animals (Basel). 2025 Oct 16;15(20):3008. doi: 10.3390/ani15203008 (PMC12560933; doi:10.3390/ani15203008)
Supplement: Supplementary file 1 [file animals-15-03008-s001.zip › animals-3862863-supplementary.pdf]

Table S1. Breeding value of bucks for milk production traits estimated using the animal model and the sire model (top 20 ranked).

N	animal model							sire model						
	**Bucks**	**TMY**	**R**	**FY**	**R**	**PY**	**R**	**Bucks**	**TMY**	**R**	**FY**	**R**	**PY**	**R**
**1**	**7410**	101.97	**1**	1.14	11	2.50	3	**7410**	78.72	**1**	0.90	9	1.82	7
**2**	**0620**	78.83	**2**	0.73	18	1.14	8	**3254**	69.52	**2**	2.86	1	2.60	5
**3**	**1148**	76.49	**3**	3.11	3	3.20	1	**1110**	56.76	**3**	1.91	3	1.77	8
**4**	**6443**	65.61	**4**	2.46	4	2.09	5	**0620**	55.42	**4**	0.68	14	0.85	14
**5**	**3254**	60.36	**5**	4.06	1	2.19	4	**4510**	49.58	**5**	1.89	4	2.78	3
**6**	**1110**	59.81	**6**	2.25	5	1.72	6	**2210**	33.62	**6**	1.30	7	0.67	16
**7**	**4450**	57.71	**7**	0.19	24	-0.01	22	**4448**	32.42	**7**	0.71	13	2.64	4
**8**	**2210**	46.37	**8**	1.95	7	1.14	7	**4510**	22.85	**8**	0.78	10	1.49	9
**9**	**4448**	46.02	**9**	0.91	14	0.70	16	**1148**	16.94	**9**	1.63	5	3.44	2
**10**	**4447**	45.08	**10**	1.46	8	0.83	12	**8510**	15.46	**10**	0.20	20	0.18	21
**11**	**9749**	44.69	**11**	-0.37	29	-0.28	28	**1230**	15.46	**11**	0.37	17	0.72	15
**12**	**6441**	39.00	**12**	0.99	13	0.81	14	**7237**	14.72	**12**	1.58	6	1.29	11
**13**	**6450**	32.95	**13**	3.95	2	2.95	2	**6443**	14.62	**13**	0.76	12	2.45	6
**14**	**1141**	29.09	**14**	2.18	6	0.97	10	**1143**	12.69	**14**	0.06	22	-0.41	26
**15**	**8510**	26.27	**15**	0.45	22	0.82	13	**4450**	9.45	**15**	-0.74	32	-1.18	35
**16**	**4510**	24.33	**16**	1.11	12	0.67	17	**6450**	8.83	**16**	2.10	2	4.76	1
**17**	**4510**	23.83	**17**	0.79	15	0.93	11	**3110**	6.24	**17**	0.33	18	0.08	22
**18**	**3110**	19.33	**18**	0.38	23	0.37	19	**6441**	5.47	**18**	0.17	21	1.38	10
**19**	**6445**	17.29	**19**	0.75	16	0.35	20	**9749**	5.14	**19**	-0.68	28	-1.22	36
**20**	**6439**	16.32	**20**	1.36	10	0.81	15	**5960**	2.62	**20**	1.14	8	0.19	20

TMY - total milk yield per lactation; FY - milk fat yield; PY - milk protein yield; R – rang;
